# Ultrasonographic diagnosis in rare primary cervical cancer

**DOI:** 10.1136/ijgc-2021-002860

**Published:** 2021-10-28

**Authors:** Jiaoling Li, Congmin Gu, Haiqing Zheng, Xiuping Geng, Zhonghan Yang, Lin Zhou, Haiying Wu

**Affiliations:** 1 Department of Ultrasound, Guangzhou Women and Children's Medical Center, Guangzhou, Guangdong Province, China; 2 Pathological Diagnosis Department, Guangzhou Women and Children's Medical Center, Guangzhou Medical University, Guangzhou, Guangdong Province, China; 3 Data Center of Pediatrics Research Institute, Guangzhou Women and Children's Medical Center, Guangzhou Medical University, Guangzhou, Guangdong Province, China; 4 Department of Biochemistry, School of Medicine, Sun Yat-sen University, Guangzhou, Guangdong Province, China; 5 Department of Gynecology and Obstetrics, Guangzhou Women and Children's Medical Center, Guangzhou Medical University, Guangzhou, Guangdong Province, China

**Keywords:** cervical cancer, adenocarcinoma, uterine cervical neoplasms, cervix uteri, genitalia, female

## Abstract

**Introduction:**

Although ultrasonography has been reported to have similar diagnostic accuracy to magnetic resonance imaging, it is not a standard imaging modality for cervical cancer. We aimed to summarize the ultrasonographic features of rare primary cervical cancer.

**Methods:**

This was a retrospective study of patients with cervical cancer who were diagnosed between June 2014 and October 2019. They were divided into common-type cervical cancer (ie, cervical squamous cell carcinoma) and rare-type cervical cancer groups including adenocarcinoma, adenosquamous carcinoma, and small cell carcinoma. All patients were staged according to the tumor, nodes, and metastases criteria.

**Results:**

Of the 64 patients, the diagnosis was suspected on ultrasonography in 61 (95.3%) patients and missed on ultrasonography in three patients. The tumor size was smaller in the rare-type cervical cancer group (p<0.05). Hypoechoic lesions in common-type cervical cancer and isoechoic lesions accounted for 74.4% (32/43) and 61.9% (13/21) of patients in the rare-type cervical cancer group, respectively (p<0.001). Meanwhile, 67.4% (29/43) of tumors in common-type cervical cancer were exophytic, while 66.7% (14/21) in rare-type cervical cancer were endophytic (p=0.01). Color Doppler blood signals, as compared with normal cervical tissue, were found in all patients. There was good consistency between ultrasonographic and pathologic diagnosis of rare-type cervical cancer (weighted kappa=0.87).

**Conclusions:**

Most patients with rare-type cervical cancer present with isoechoic lesions. The coincidence rate between ultrasonographic and pathologic diagnosis of rare-type cervical cancer is 87%.

**HIGHLIGHTS**

- Rare-type cervical cancer has a lower tumour volume than the more common-type cervical cancer
- Most patients with rare-type cervical cancer have endophytic tumour growth and isoechoic lesions
- Ultrasound may be used for detecting rare-type cervical cancer for early diagnosis

## INTRODUCTION

Squamous cell carcinoma is the most common primary tumor in the cervix. Other rare primary tumors include clear-cell carcinoma and small-cell carcinoma,^1–3^ primary lymphoma,^4^ cervical choriocarcinoma,^5^ sarcoma of the uterine cervix,^6^ malignant melanoma,^7^ Wilm’s tumor,^8^ and malignant peripheral neurilemmoma of cervical fibroblasts.^9^ Cervical cancer primarily presents with hemorrhage in the early disease stage. Meanwhile, late clinical manifestations include irregular vaginal bleeding of varying amounts with corresponding symptoms of distant metastasis.

Pap smears are used for primary prevention to screen for pre-invasive lesions, enabling early diagnosis and treatment that can lead to significantly lower mortality rates. However, this method is inadequate for women with rare cervical cancers, such as small-cell carcinoma of the cervix, that do not invade the surface epithelium of the cervix but diffusely infiltrate cervical stroma.^10^ In these patients, cytological examination results are often negative. The human papillomavirus (HPV) test has recently been added to the Pap smear as a screening modality for cervical cancer in some countries.^11 12^ Further, HPV vaccines have been introduced to prevent cervical cancer.^13^

Ultrasonography is a cost-effective, convenient, and non-invasive modality with a diagnostic accuracy similar to that of magnetic resonance imaging (MRI) for evaluating local extension of cervical cancer; however, it is not considered a standard diagnostic modality.^14 15^ Ultrasonography may be suitable for women with rare cervical cancers. Specifically, color Doppler ultrasonography can enhance the display rate of malignant tumors by Doppler flow imaging and obtain clearer images for accurate staging and targeted cervical biopsy. These features are helpful for improving the diagnostic sensitivity and positive predictive value of ultrasonography as well as providing a basis for treatment planning. Our study investigated different ultrasonographic features of rare primary and common cervical cancer and examined the diagnostic and staging accuracy of ultrasonography for such cancers.

## METHODS

### Study Design and Patients

This study was approved by the appropriate ethics review board (approval number: 54.4 25; 6 January 2014). All patients with clinical concern for cervical cancer provided informed consent for ultrasonography assessment and underwent clinical examination, HPV testing, and cervical biopsy after ultrasound and before treatment.

In this retrospective study of patients with cervical cancer at our hospital between June 2014 and October 2019, patient records were searched for cervical cancer using the keywords ‘abnormal echogenicity of cervix’ and ‘suspicious cervical cancer’ in the Image Workstation and ‘cervical cancer’ in the Pathology Specimen Bank. A total of 2 862 285 patients underwent ultrasonography during the study period. Only 65 patients had suspicion of cervical cancer on ultrasonography and underwent cervical biopsy, and four were misdiagnosed by ultrasonography and were excluded from the study. Three patients did not have suspicion of cervical cancer on ultrasonography but were confirmed as having cervical cancer via pathology and were included in the study. Thus, 64 patients with cervical cancer were included in the analyses. They were divided into common-type cervical cancers (n=43 patients) and rare-type cervical cancers (n=21 patients).

### Ultrasonography Assessment

A high-resolution ultrasonography system (Voluson E8 Expert; GE Healthcare, USA) equipped with a 3–5 MHz convex array transducer and a 7–12 MHz vaginal transducer was used to detect cervical cancer. All data and images were recorded according to the last ultrasound performed 5 days before treatment. The ultrasound technique was as follows. First, on two dimensions, the uterus, endometrium, cervix, vagina, bilateral parametrium, and bilateral appendages were observed. Echogenicity (hypoechoic, isoechoic, hyperechoic, and mixed compared with the surrounding tissue) of cervical lesions was described. Three standardized diameters of primary cervical cancer (craniocaudal and anteroposterior in a longitudinal projection from the outmost margin of the lesion to the most cranial extension of the lesion and perpendicular to it, respectively, and transverse diameter in the transverse projection from the outmost lateral aspects of the lesion) were measured when cervical cancer was suspected.

Tumor volume was calculated using the ellipsoid formula $A\times B\;\times C\;\times \frac{\pi }{6}$ ,^16^ where *A*, *B*, and *C* were diameters of the tumor (craniocaudal, anteroposterior, and laterolateral measurements, respectively). Blood flow signal distribution in and around the cervical lesions was observed using color Doppler flow imaging. Next, abnormal echogenicity of lesions and Doppler flow signals (compared with that of adjacent normal tissue) were used to judge whether adjacent tissues (including the corpus, parametrial tissue, bilateral appendages, bladder, and rectum) were infiltrated. For patients with infiltration, infiltration depth was also assessed. First-grade group lymph nodes were then observed. Next, growth patterns were judged and classified as exophytic (papillary growth) and endophytic (the lesion infiltrating into deep cervical tissue). Finally, all patients were staged according to tumor, node, and metastases criteria. Vascularization was classified subjectively using a ‘color score’ as follows: punctate blood flow signals, score=1; short rod blood flow signals, score=2; strip blood flow signals, score=3; and reticular blood flow signals, score=4. Meanwhile, 40 patients without metal objects in their body underwent MRI (Signa; GE Medical Systems, Milwaukee, Wisconsin, USA) to assist with staging. Patients with suspected cervical cancer underwent targeted cervical biopsy. All patients with confirmed cervical cancer underwent primary surgery, pre-operative or post-operative chemotherapy, or radiation therapy depending on the diagnosis and the standard treatment recommendations.

### Statistical Analysis

Data are presented as mean±SD. Between-group comparisons were conducted using the Student’s t-test, two-tailed Fisher’s exact probability test, χ^2^ tests, or Mann–Whitney U test, as appropriate. Weighted kappa was used to evaluate the consistency between ultrasonography and pathologic diagnosis. All statistical analyses were performed using SAS software Version 9.4 (SAS, Cary, North Carolina, USA). A two-sided p value of <0.05 was considered statistically significant.

## RESULTS

**Figure 1:**
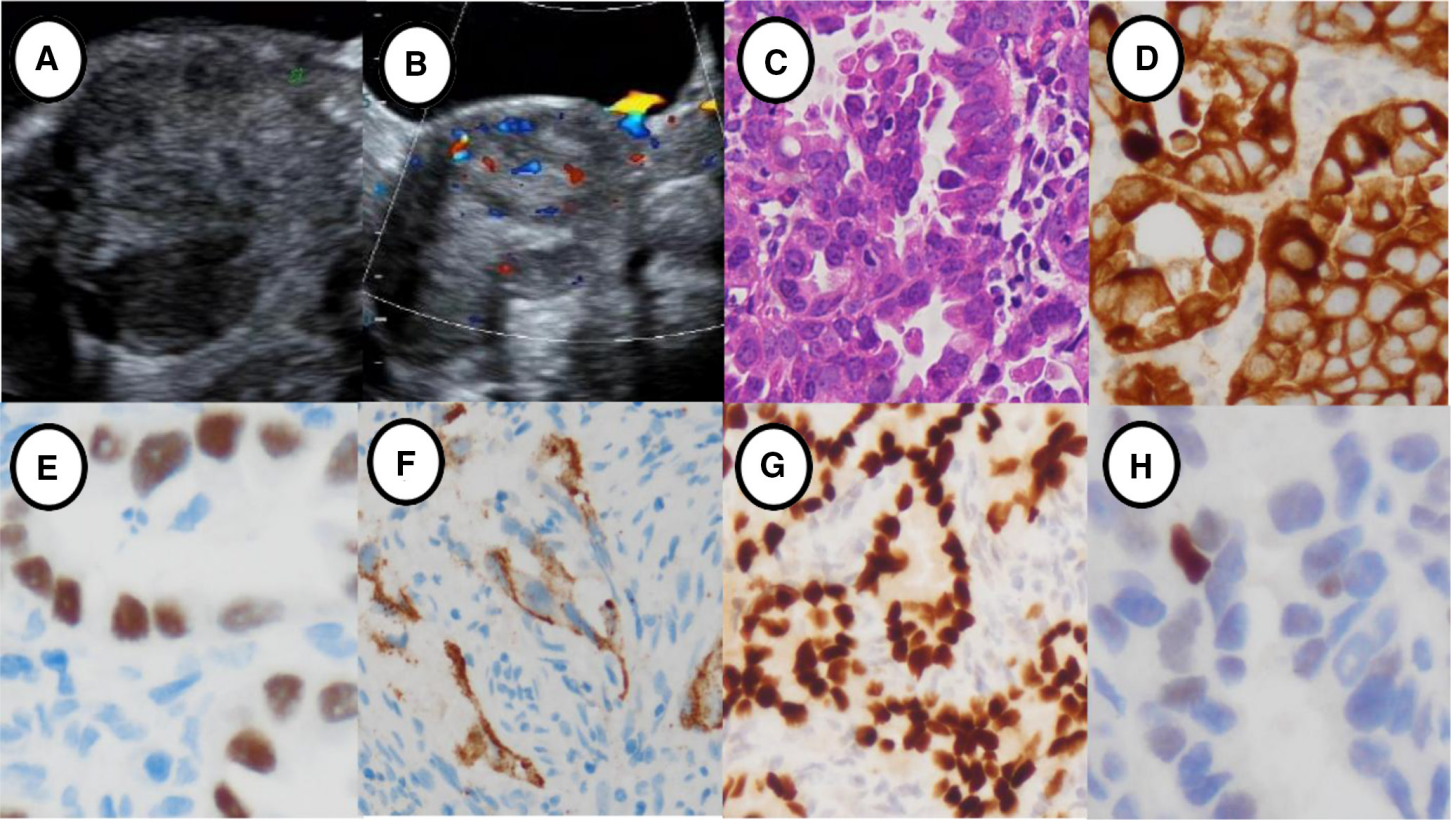
Ultrasonographic features and immunohistochemical staining of primary clear-cell carcinoma of the cervix in an unmarried celibate woman. (A) Two-dimensional ultrasonography showing a hypoechoic cervical lesion. (B) Color Doppler ultrasonography showing significant short rod blood flow signals in the lesion. (C) Pathological features of clear-cell carcinoma of the cervix (HE, ×20). (D) CK7 was positive in clear-cell carcinoma of the cervix (×20). (E) HNF was positive (×20). (F) Napsin A was positive (×20). (G) PAX was positive (×10). (H) P53 was positive (×20).

**Figure 2:**
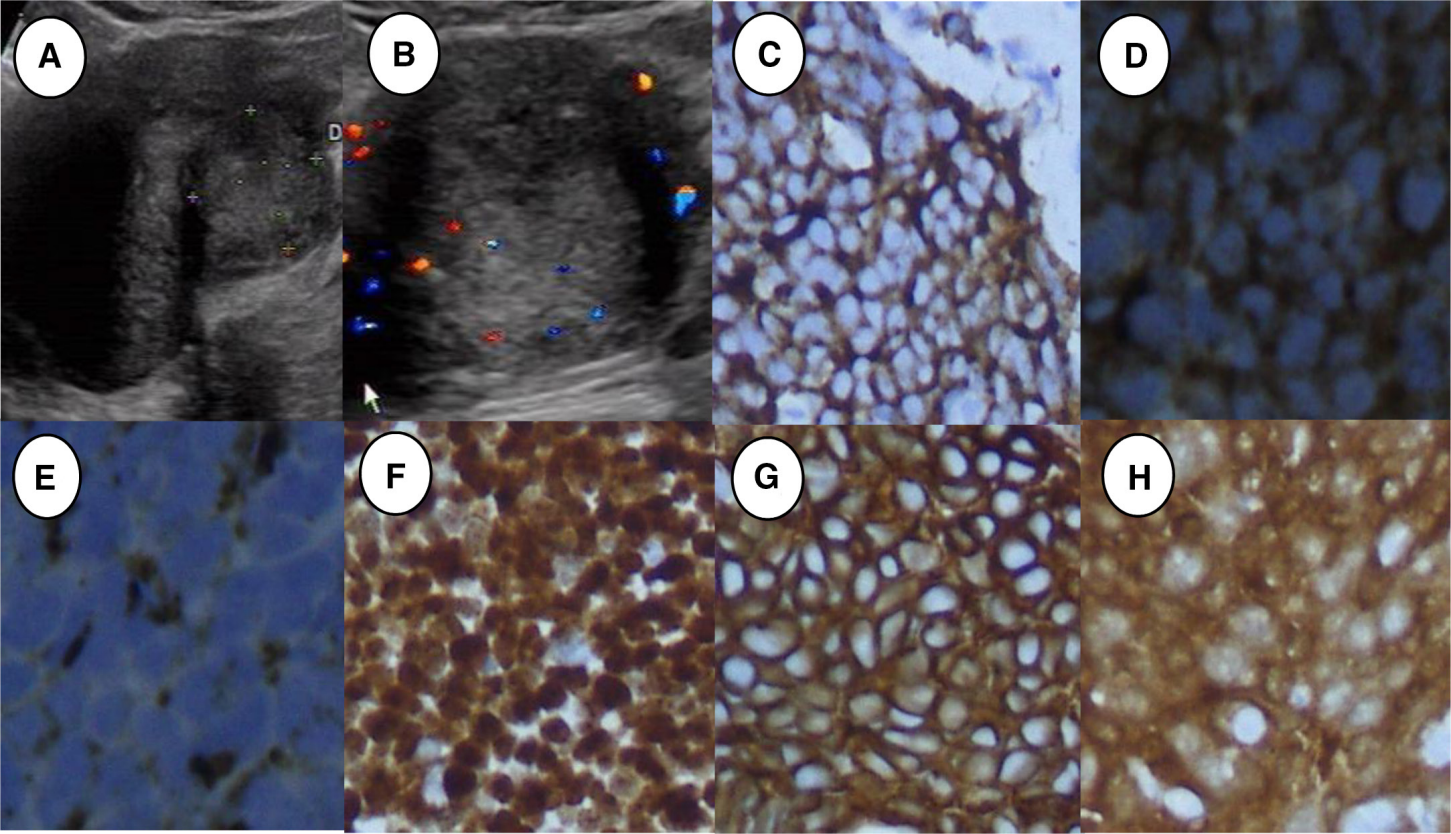
Ultrasonographic features and immunohistochemical staining of primary small-cell carcinoma of the cervix in a sexually active married woman. (A) Two-dimensional ultrasonography showing an isoechoic cervical lesion. (B) Color Doppler ultrasonography showing significant strip blood flow signals in the lesion. (C) Syn was positive (×20). (D) NSE was positive (×40). (E) CGA was positive (×40). (F) Ki-67 was positive (×20). (G) CEA was positive (×20). (H) P16 was positive (×20).

The average age of patients in the common-type and rare-type cervical cancer groups was 51.05±9.41 years (range 31–69 years) and 48.05±9.67 years (range 23–64 years), respectively. Regarding the pathological results, 64 patients were diagnosed with cervical cancer. All 43 patients in the common-type cervical cancer group had cervical squamous cell carcinoma (one patient had squamous cell carcinoma in situ). The rare-type cervical cancer group included 15 patients with adenocarcinoma (one patient with clear-cell carcinoma), two patients with adenosquamous carcinoma, and four patients with small-cell carcinoma. The patient with clear-cell carcinoma stained positively for Napsin A, HNF-1B, CK7, PAX, and P53 by immunohistochemistry staining. Meanwhile, the patients with small-cell carcinoma had positive immunohistochemical staining for Syn, chromogranin A, neuron-specific enolase, Ki-67, CEA, and P16 (Figures 1 and 2).

Regarding the ultrasonographic results (using pre-operative ultrasonography), 65 patients were suspected of having cervical cancer. Of these, two patients with chronic cervicitis and two with submucosal myoma were misdiagnosed with cervical cancer. Furthermore, the diagnoses of three patients with clinical symptoms were missed on ultrasonography but confirmed by pathology. The sensitivity and the positive predictive value of ultrasonography for diagnosis was 95.31% (61/64) and 93.85% (61/65), respectively. Regarding the diagnosis according to the tumor, nodes, and metastases, ultrasonography was used to diagnose 27 patients with stage T1b1 disease, seven patients with stage T1b2 disease, five patients with T2a1 disease, 13 patients with stage T2a2 disease, three patients with stage T2b disease, one patient with stage T3a disease, and eight patients with stage T4 disease. The general demographic information, ultrasonographic and histological characteristics, and HPV infection of 43 patients in the common-type cervical cancer group and of 21 patients in the rare-type cervical cancer group are shown in Table 1 and Figures 1 and 2. HPV infection rates were 83.7% (36/43) and 47.6% (10/21) in common-type and rare-type cervical cancer, respectively. Color Doppler ultrasonography showed sparse or dense punctate blood flow signals (score=1) in seven patients, short rod blood flow signals (score=2) in nine patients, strip blood flow signals (score=3) in 16, and reticular blood flow signals (score=4) in 32 (online supplemental file 1).

10.1136/ijgc-2021-002860.supp1 Supplementary data

**Table 1:** General information and ultrasonographic and histological characteristics

	Common-type cervical cancer (n=43)	Rare-type cervical cancer (n=21)	T/Z/χ^2^	P value
Demographics				
DemographicsAge (mean±SD)	51.0±9.4	48.0±9.7	1.19	0.24
Ultrasonography				
Tumor size (mean±SD)				
Craniocaudal (mm)	39.58±12.94	31.62±14.06	2.25	0.03
Anteroposterior (mm)	29.35±10.90	23.05±10.96	2.17	0.03
Laterolateral (mm)	29.70±9.61	24.00±11.03	2.12	0.04
Volume (cm^3^), median (IQR)	17.89 (9.96, 34.14)	10.61(1.77, 19.56)	−2.17	0.03*
Echogenicity, n (%)			–	<0.001†§
Hypoechoic	32 (74.42)	5 (23.81)		
Hyperechoic	2 (4.65)	0		
Isoechoic	3 (6.98)	13 (61.90)		
Mixed echoic	6 (13.95)	3 (14.29)		
Hysterocele, n (%)			–	0.67†
No	35 (81.39)	16 (76.19)		
Yes	7 (16.28)	5 (23.81)		
Pregnancy	1 (2.33)	0		
Color score‡				
1	2 (4.65)	5 (23.81)		
2	6 (13.95)	3 (14.29)		
3	12 (27.90)	4 (19.05)		
4	23 (53.50)	9 (42.85)		
Grade, n (%)			–	0.51†
Poorly differentiated	7 (16.28)	6 (28.57)		
Moderately differentiated	26 (60.47)	11 (52.38)		
Well differentiated	10 (23.26)	4 (19.05)		
Growth pattern, n (%)			6.67	0.01
Endophytic	14 (32.56)	14 (66.67)		
Exophytic	29 (67.44)	7 (33.33)		
HPV infection, n (%)				
No	7 (16.28)	11 (52.38)	9.1	0.003
Yes	36 (83.72)	10 (47.62)		

*Mann-Whitney U-test (rank-sum test of non-normal distribution data).

†Two-tailed Fisher’s exact test (expected frequency <1, accurate probability method).

‡Color score is a subjective estimation of the vascularity within the tumor (–, without value).

§Echogenicity compared with surrounding cervical tissue.

¶Data are presented as mean±SD or n (%). Other unmarked variables adopt Student’s t-test or χ^2^ tests.

HPV, human papillomavirus.

Pathologic results showed that lesions were limited to the cervix in 31 (48.4%) patients and had infiltrated adjacent local tissues in 33 (51.6%). Local lymph node metastases occurred in seven patients (16.3%, 7/43) in the common-type cervical cancer group and three patients (14.3%, 3/21) in the rare-type cervical cancer group. In addition, intravascular cancer thrombi were found in five patients (11.6%, 5/43) and two patients (9.5%, 2/21) in the common-type and rare-type cervical cancer (small-cell carcinoma) groups, respectively. Analysis of weighted kappa showed that the consistency between ultrasonography and pathologic diagnosis of rare-type cervical cancer was high (weighted kappa=0.87 (95% CI 0.65 to 1.00)) (Table 2).

**Table 2:** Staging consistency of ultrasonography and pathology in rare-type cervical cancer

USG	Pathology									
	Tis	T1b1	T1b2	T2a1	T2a2	T2b	T3a	T3b	T4	Total
Tis	0	0	0	0	0	0	0	0	0	0
T1b1	0	11	0	0	0	0	0	0	0	11
T1b2	0	0	3	0	0	0	0	0	0	3
T2a1	0	0	0	2	0	0	0	0	0	2
T2a2	0	0	0	0	2	0	0	0	0	2
T2b	0	0	0	0	0	1	0	0	0	1
T3a	0	0	0	0	0	0	0	0	0	0
T3b	0	0	0	0	0	0	0	0	0	0
T4	0	0	0	1	0	0	0	0	1	2
Total	0	11	3	3	2	1	0	0	1	21

Weighted kappa=0.87(95% CI 0.65 to 1.00). For the convenience of statistics, T2a2N1 was marked as T2a2; T2bN2 was marked as T2b; T3aN1 was marked as T3a; T3bN1 was marked as T3b.

## DISCUSSION

### Summary of Main Results

In this study we found that the sensitivity and positive predictive value of ultrasound diagnosis were 95.31% and 93.85%, respectively. Tumor volume was smaller in rare-type cervical cancer than in the more common histologic subtypes, with endophytic growth and isoechoic lesions.

### Results in the Context of Published Literature

A recent study^17^ has shown that cervical cancer lesions are mostly hypoechoic, while isoechoic, hyperechoic, and mixed echoic lesions are rare. In this study, hypoechoic lesions were found in 57.8% of patients, lower than that reported by Xu et al.^17^ However, in common-type cervical cancer, hypoechoic lesions were found in 74.5% of patients, consistent with the study by Epstein et al.^18^ Meanwhile, in rare-type cervical cancer, only five lesions (23.8%) were hypoechoic, with 13 patients (61.9%) having isoechoic lesions. This rate is consistent with that reported by Epstein et al.^18^ Furthermore, the echogenicity of lesions was significantly different between the two groups. Overall, the diagnosis in three of 13 patients with isoechoic lesions in the rare-type cervical cancer group was missed on ultrasonography, but color Doppler ultrasonography showed color flow signals in these lesions. These findings suggest that color Doppler ultrasonography is a helpful modality for diagnosing rare-type cervical cancer and may be included in standard diagnostic imaging. There was a significant difference in tumor size between the two groups. Therefore, tumor size and the echogenicity of lesions can also be used to diagnose rare-type cervical cancer.

Uterine effusion is often caused by the accumulation of fluid secreted in the uterine cavity and the obstruction of the cervical canal by the lesion. In this study, five patients (23.8%) with rare-type cervical cancer and seven patients (16.3%) with common-type cervical cancer had uterine effusion, with no significant difference between the two groups. Therefore, uterine effusion cannot be used to differentiate rare-type cervical cancer from common-type cervical cancer. In contrast, 67.4% of lesions in the common-type cervical cancer group were exophytic, while 66.7% of lesions in the rare-type cervical cancer group were endophytic, with a significant difference between the two groups (p=0.01). This confirmed that rare-type cervical cancer often diffusely infiltrates the cervical stroma.^10^

Sozzi et al found no significant difference in the detection rate of parametrial invasion on ultrasonography, MRI, and examination under anesthesia. However, the integrated pre-surgical diagnostic algorithm could better define the local extent of cervical cancer.^19^ In our study, color Doppler flow signals were found in the cancerous tissue of all patients compared with normal cervical tissue, in which no detectable vascularization was found. The coincidence rate between pre-operative ultrasonography and tumor, nodes, and metastases staging was 87% in rare-type cervical cancer, which was higher than that reported by Byun et al (62.5%) but consistent with the rate reported by Ghi et al (85.7%).^20 21^ These differences may have been caused by a local infection resulting from prolonged vaginal bleeding in patients with cervical cancer.

Cervical biopsy is the gold standard for the diagnosis of cervical cancer; however, ultrasonography is helpful for targeted cervical biopsy, especially for early diagnosis of rare-type cervical cancer. Although MRI has a high soft-tissue resolution and can be used for pre-operative staging, it is contraindicated if metal objects are present in the body.^14 16^ With improvements in the resolution achieved by ultrasonic instruments, ultrasonography may be used to determine whether the cervical line is interrupted or if the cervical intima is thickened. Moreover, ultrasonography is a cost-effective and non-invasive technique. Thus, ultrasonography is becoming the preferred modality for the early evaluation of cervical cancer. Transvaginal ultrasonography is a superior method for showing the degree of infiltration in the adjacent tissues. Furthermore, transvaginal ultrasonography is superior to MRI in determining the scope of surgery and the need for radiotherapy and chemotherapy before surgery.^15^ However, ultrasonography also has its limitations since it can only detect invasive cervical cancer.

### Strengths and Weaknesses

Our study has some limitations. All the patients were treated at our hospital, therefore selection bias may have distorted our results. In addition, no T1a1 or T1a2 tumors were included in the analysis; only macroscopic tumors were investigated. Furthermore, the study was a retrospective study and patient selection for ultrasound evaluation was not set a priori. Similarly, given the small number of patients, it is difficult to know if these results would be widely applicable to a larger population. We also did not have documentation as to the level of expertise of the ultrasonographer, nor did we examine inter- or intra-observer variability. There was also no direct comparison with MRI. Last, there was no pathology review to ascertain that the original diagnosis was in fact as stated in the medical records for the unique purposes of this study.

### Implications for Practice and Future Research

It is necessary to accumulate more patients, especially those with microscopic tumors, from multiple centers and perform a multifactor comprehensive analysis. In addition, we need to perform a prospective study which will detect rare-type cervical cancer according to the ultrasonographic features of endophytic growth, smaller tumor volume, and isoechoic lesions.

## CONCLUSIONS

In rare-type cervical cancer, most lesions are isoechoic, tumor sizes are smaller, and growth pattern is endophytic. The ultrasonographic features identified in this study can aid in the evaluation and treatment of rare-type cervical cancer, which may potentially translate to improved prognosis. Given the cost-effectiveness and potential efficacy of ultrasonography, it is worth emphasizing that it may improve diagnosis and outcomes. This may be particularly pertinent in lower income hospitals or developing nations with fewer resources where MRI is less attainable.

## Data Availability

Data are available in a public, open access repository.
