# Supplementary material for: Ultrasonographic diagnosis in rare primary cervical cancer
Source: Int J Gynecol Cancer. 2021 Oct 28;31(12):1535–40. doi: 10.1136/ijgc-2021-002860 (PMC8666808; doi:10.1136/ijgc-2021-002860)
Supplement: Supplementary data [file ijgc-2021-002860supp001.pdf]

	CTCC	craniocaudal (mm)	anteroposterior (mm)	laterolateral (mm)
1	case1	51	44	40
2	case2	43	39	37
3	case3	24	10	20
4	case4	47	32	37
5	case5	33	32	20
6	case6	45	42	41
7	case7	28	24	25
8	case8	18	11	13
9	case9	34	34	34
10	case10	44	43	36
11	case11	35	13	26
12	case12	46	18	23
13	case13	48	34	40
14	case14	29	29	29
15	case15	38	36	27
16	case16	60	49	30
17	case17	21	20	20
18	case18	72	31	12
19	case19	37	26	36
20	case20	50	29	28
21	case21	42	33	41
22	case22	40	31	39
23	case23	19	18	16
24	case24	38	36	25
25	case25	50	40	36
26	case26	33	16	23
27	case27	33	22	28
28	case28	74	48	44
29	case29	50	40	36
30	case30	40	40	36
31	case31	35	31	30
32	case32	49	36	42
33	case33	33	21	29
34	case34	49	26	31
35	case35	33	25	26
36	case36	38	17	36
37	case37	52	37	35
38	case38	27	26	13
39	case39	36	21	33
40	case40	15	7	12
41	case41	38	30	25
42	case42	21	16	15
43	case43	54	49	52

	RTCC			
1	case1	47	28	29
2	case2	25	20	14
3	case3	34	21	29
4	case4	65	41	41

5	case5	34	25	22
6	case6	50	35	44
7	case7	30	22	29
8	case8	44	44	30
9	case9	40	22	26
10	case10	30	26	26
11	case11	30	28	28
12	case12	40	36	26
13	case13	45	36	45
14	case14	21	6	14
15	case15	34	24	28
16	case16	10	10	10
17	case17	12	12	6
18	case18	15	15	15
19	case19	21	12	19
20	case20	15	11	13
21	case21	22	10	10

volume（cm3）	Sexual history	Marital history	age	Hysterocele	well differentiated
46.94	yes	yes	51	no	well differentiated
32.45	yes	yes	55	no	well differentiated
2.51	yes	yes	69	no	well differentiated
29.1	yes	yes	62	no	
11.05	yes	yes	61	no	well differentiated
40.53	yes	yes	53	no	
8.79	yes	yes	49	no	
1.35	yes	yes	51	no	
20.56	yes	yes	54	no	
35.62	yes	yes	66	no	
4.42	yes	yes	38	no	
9.96	yes	yes	37	no	
34.14	yes	yes	57	yes	
12.76	yes	yes	55	no	
19.32	yes	yes	39	no	
46.13	yes	yes	54	no	well differentiated
4.39	yes	yes	35	no	
14.01	yes	yes	67	yes	
18.11	yes	yes	50	no	
21.23	yes	yes	53	no	
29.72	yes	yes	42	no	
25.29	yes	yes	63	no	well differentiated
2.86	yes	yes	49	no	
17.89	yes	yes	50	no	
37.66	yes	yes	67	yes	well differentiated
6.35	yes	yes	52	no	well differentiated
10.63	yes	yes	60	no	
81.74	yes	yes	52	no	
37.66	yes	yes	51	no	
30.12	yes	yes	52	no	
17.02	yes	yes	54	yes	
38.75	yes	yes	53	no	
10.51	yes	yes	46	no	
20.66	yes	yes	54	no	well differentiated
11.22	yes	yes	49	yes	
12.16	yes	yes	43	no	
35.22	yes	yes	55	yes	
4.77	yes	yes	32	no	
13.05	yes	yes	43	no	
0.66	yes	yes	31	pregnancy	
14.91	yes	yes	41	no	well differentiated
2.64	yes	yes	40	no	
71.96	yes	yes	60	yes	
19.96	yes	yes	50	yes	
3.66	yes	yes	55	yes	
10.83	yes	yes	49	no	well differentiated
57.15	yes	yes	54	no	

9.78	yes	yes	51	no	
40.27	yes	yes	64	yes	
10.01	yes	yes	60	yes	well differentiated
30.38	yes	yes	62	no	well differentiated
11.97	No	No	23	no	
10.61	yes	yes	47	no	
12.3	yes	yes	45	no	
19.56	yes	yes	58	yes	
38.13	yes	yes	44	no	
0.92	yes	yes	42	no	
11.95	yes	yes	48	no	
0.52	yes	yes	55	no	
0.45	yes	yes	44	no	
1.77	yes	yes	35	no	
2.5	yes	yes	41	no	
1.12	yes	yes	39	no	well differentiated
1.15	yes	yes	43	no	

Grade		Echogenicity		
moderately differentiated	poorly differentiated	Hypoechoic	Hyperechoic	Isoechoic
		Hypoechoic		
		Hypoechoic		
		Hypoechoic		
moderately differentiated		Hypoechoic		
		Hypoechoic		
moderately differentiated				
moderately differentiated		Hypoechoic		
moderately differentiated		Hypoechoic		
	poorly differentiated	Hypoechoic		
moderately differentiated				Isoechoic
moderately differentiated		Hypoechoic		
moderately differentiated		Hypoechoic		
moderately differentiated		Hypoechoic		
	poorly differentiated	Hypoechoic		
moderately differentiated		Hypoechoic		
		Hypoechoic		
	poorly differentiated	Hypoechoic		
moderately differentiated			Hyperechoi	
moderately differentiated		Hypoechoic		
moderately differentiated				Isoechoic
moderately differentiated		Hypoechoic		
		Hypoechoic		
moderately differentiated				Isoechoic
	poorly differentiated	Hypoechoic		
		Hypoechoic		
moderately differentiated		Hypoechoic		
	poorly differentiated	Hypoechoic		
moderately differentiated			Hyperechoi	
	poorly differentiated			
	poorly differentiated			
moderately differentiated		Hypoechoic		
moderately differentiated		Hypoechoic		
moderately differentiated		Hypoechoic		
moderately differentiated		Hypoechoic		
moderately differentiated		Hypoechoic		
moderately differentiated		Hypoechoic		
moderately differentiated		Hypoechoic		
moderately differentiated		Hypoechoic		
moderately differentiated		Hypoechoic		
moderately differentiated		Hypoechoic		
moderately differentiated				
moderately differentiated		Hypoechoic		
		Hypoechoic		
	poorly differentiated	Hypoechoic		

moderately differentiated			Isoechoic
moderately differentiated			Isoechoic
			Isoechoic
	poorly differentiated	Hypoechoic	
	poorly differentiated		Isoechoic
	poorly differentiated		Isoechoic
	poorly differentiated	Hypoechoic	
	poorly differentiated		
moderately differentiated			Isoechoic
moderately differentiated			Isoechoic
moderately differentiated			Isoechoic
moderately differentiated			Isoechoic
moderately differentiated			Isoechoic
moderately differentiated			Isoechoic
			Isoechoic
moderately differentiated			Isoechoic

	Growth pattern		HPV infection		宫颈原位癌	T1a(宫颈癌)
Mixed echoic	Exophytic	Endophytic	positive	negative	Tis	T1a1
	Exophytic			negative		
	Exophytic		positive			
	Exophytic		positive			
	Exophytic		positive			
	Exophytic		positive			
Mixed echoic	Exophytic			negative		
	Exophytic		positive			
	Exophytic			negative		
	Exophytic		positive			
		Endophytic	positive			
	Exophytic			negative		
	Exophytic		positive			
		Endophytic	positive			
		Endophytic	positive			
	Exophytic		positive			
	Exophytic		positive			
	Exophytic		positive			
		Endophytic	positive			
		Endophytic	positive			
	Exophytic		positive			
	Exophytic			negative		
	Exophytic		positive			
	Exophytic		positive			
		Endophytic	positive			
		Endophytic	positive			
Mixed echoic	Exophytic			negative		
	Exophytic		positive			
	Exophytic		positive			
		Endophytic	positive			
Mixed echoic		Endophytic	positive			
Mixed echoic		Endophytic	positive			
		Endophytic	positive			
		Endophytic	positive			
Mixed echoic	Exophytic		positive			
	Exophytic		positive			
	Exophytic		positive			
	Exophytic		positive			
	Exophytic		positive			
		Endophytic	positive			
	Exophytic		positive			
Mixed echoic	Exophytic		positive			
	Exophytic		positive			
		Endophytic		negative		
Mixed echoic		Endophytic		negative		
		Endophytic		negative		
		Endophytic		negative		
	Exophytic			negative		

	Exophytic		positive	
		Endophytic	positive	
Mixed echoic		Endophytic		negative
		Endophytic		negative
		Endophytic		negative
	Exophytic		positive	
	Exophytic		positive	
	Exophytic		positive	
Mixed echoic		Endophytic	positive	
		Endophytic	positive	
	Exophytic			negative
	Exophytic		positive	
		Endophytic	positive	
		Endophytic		negative
		Endophytic		negative
		Endophytic	positive	
		Endophytic		negative

					TNM
局限于子宫）	T1b（宫颈癌局限于子宫）		T2a无宫旁浸润		T2b有宫旁浸润
T1a2	T1b1	T1b2	T2a1	T2a2	T2b
		T1b2			
				T2a2	
			T2a1		
				T2a2	
			T2a1		
				T2a2	
	T1b1				
	T1b1				
	T1b1				
	T1b1				
				T2a2	
	T1b1				
	T1b1				
		T1b2			
	T1b1				
		T1b2			
	T1b1				
				T2a2	
					T2b
			T2a1		
				T2a2	
	T1b1				
	T1b1				
				T2a2	
		T1b2			
				T2a2	
					T2b
				T2a2	
	T1b1				
				T2a2	
	T1b1				
				T2a2	
	T1b1				
	T1b1				
	T1b1				
	T1b1				
				T2a2	
	T1b1				
	T1b1				
				T2a2	

T1b1
T1b2
T2a1
T1b2
T2b

T2a1
T1b2

T1b1
T1b1
T1b1
T1b1
T1b1
T1b1
T1b1
T1b1

T3a（侵及下1/3阴道，未到达盆壁	T3b（到达盆壁或引起肾积水或无功能肾	T4		
T3a	T3b	T4	Tis	T1a1
		T4		
		T4		
		T4		
		T4		
T3a				
		T4		
			Tis	
		T4		

T4

T4

	pathology							
T1a2	T1b1	T1b2	T2a1	T2a2	T2b	T3a	T3b	T4
		T1b2						
						T3a		
			T2a1					
				T2a2				
			T2a1					
				T2a2N1				
			T2a1					
	T1b1							
	T1b1N1							
								T4
				T2a2				
				T2a2				
								T4
	T1b1							
	T1b1							
		T1b2						
	T1b1							
		T1b2						
	T1b1							
				T2a2				
					T2bN1			
								T4
								T4
							T3bN1	
				T2a2				
	T1b1							
	T1b1							
				T2a2				
		T1b2						
				T2a2				
							T3bN1	
						T3aN1		
	T1b1							
				T2a2				
	T1b1							
						T3a		
						T3a		
	T1b1N1							
			T2a1					
	T1b1							
	T1b1							
								T4
				T2a2N1				
	T1b1							
	T1b1							
				T2a2				

T1b1
T1b2N1
T2a1
T1b2
T2b
T2a1N1
T2a1
T1b2
T4
T1b1
T1b1
T1b1
T1b1
T1b1
T1b1
T1b1
T1b1
